# Bell’s palsy: data from a study of 70 cases

**Published:** 2014

**Authors:** D Cirpaciu, CM Goanta

**Affiliations:** * Alexandria County Emergency Hospital, Romania; ** ”Carol Davila” University of Medicine and Pharmacy, Bucharest, Romania

**Keywords:** Bell’s palsy, facial nerve, statistical data

## Abstract

Bell’s palsy is a condition that affects the facial nerve, which is one of the twelve cranial nerves. Its main function is to control all the muscles of the facial expression. It is a unilateral, acute, partial or complete paralysis of the facial nerve. Bell's palsy remains the most common cause of facial nerve paralysis, more often encountered in females aged 17 to 30 years, recurrent in many cases and with poor associations with other pathologic conditions. In modern literature, the suspected etiology could be due to the reactivation of the latent herpes viral infections in the geniculate ganglia, and their subsequent migration to the facial nerve but, favorable outcome by using vasodilators, neurotrophic and corticosteroid therapy was recorded.

## Introduction

Bell’s palsy is a condition that affects the facial nerve, which is one of the twelve cranial nerves. Its main function is to control all the muscles of the facial expression. It is a unilateral, acute, partial or complete paralysis of the facial nerve without other neurologic complains [**2**]. Bell's palsy is a common but still controversial disease, with unknown etiology until now, which raises important aesthetic and psychological issues because it involves the ability to express emotion by controlling the position of the mouth, nostrils, and eyebrows, and control the eye closure, drooling and speech. Regarding therapy, clinical trials showed a significant benefit from treating Bell's palsy with corticosteroids [**4**,**5**,**10**,**11**]. According to the most reliable studies, antivirals have not proved superior to placebo [**3**]. The surgical treatment is controversial [**11**], and there are few controlled clinical trials for the effectiveness of physical therapies, massage and facial exercises. In most studies, the incidence oscillates between 11 and 40 cases per 100,000 inhabitants per year [**1**,**6**,**9**]. Even though Bell’s palsy is a common disease, there are few statistical data in the medical literature and lack Romanian data.

**Purpose:** An attempt to analyze some clinical and epidemiologic aspects of Bell’s palsy, to offer statistic information about this disease, and to develop relevant correlations between the existing data in literature and those obtained in this study. 

## Materials & Methods

• a sample of 86 patients admitted in “Professor Dr. Dorin Hociota” Institute of Phonoaudiology and Functional ENT Surgery, Bucharest, Romania, between January 2005 and December 2009, were analyzed,

• patients between 17 and 79 years old with idiopathic facial paralysis were included,

• cases with paralysis secondary to trauma, chronic or acute otitis media, otomastoiditis, zoster oticus, cerebral stroke, or other condition affecting the ear and parotid gland were excluded,

• they were divided according to demographic parameters, treatment and related diseases,

• the programs used were the following: Excel 2007 for graphics.

## Results

**Fig. 1:**
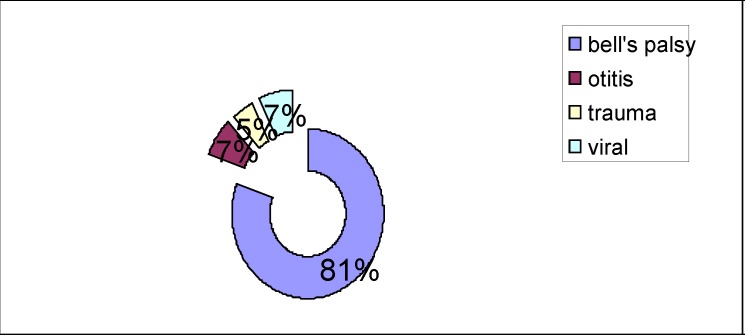
Causes of facial paralysis

Of the 86 patients admitted, suffering from partial or complete facial paralysis, 70 were diagnosed with idiopathic Bell's palsy, six cases with herpes zoster oticus, four cases with traumatic facial palsy and six patients had chronic or acute otitis media (**Fig. 1**).

**Fig. 2:**
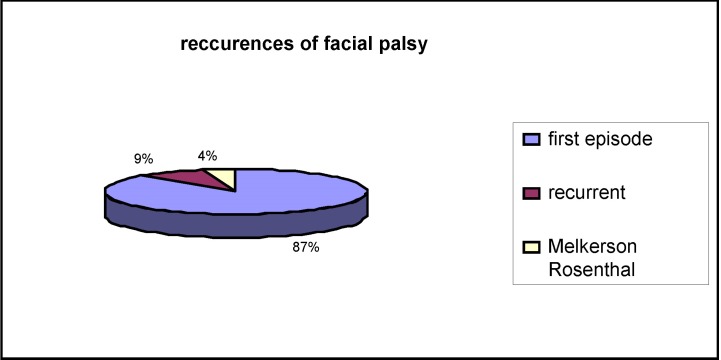
Recurrences of facial palsy

Of the 70 patients with Bell’s palsy, 6 had recurrent facial palsy and three were considered to have Melkerson Rosenthal syndrome. Some authors reported a higher risk for pregnant women to develop idiopathic facial palsy [**7**,**10**], but none of our 70 cases of Bell’s palsy was found during pregnancy or puerperium (**Fig. 2**).

**Fig. 3:**
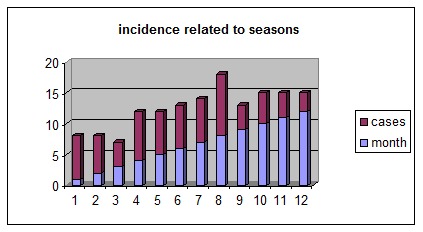
Incidence related to seasons

In the study, peripheral facial palsy was more frequent in the warm period (spring and summer) with a peak of incidence in august (**Fig. 3**), in contrast with the results of other studies in literature, that found a decreasing frequency in warm weather [**8**].

**Fig. 4:**
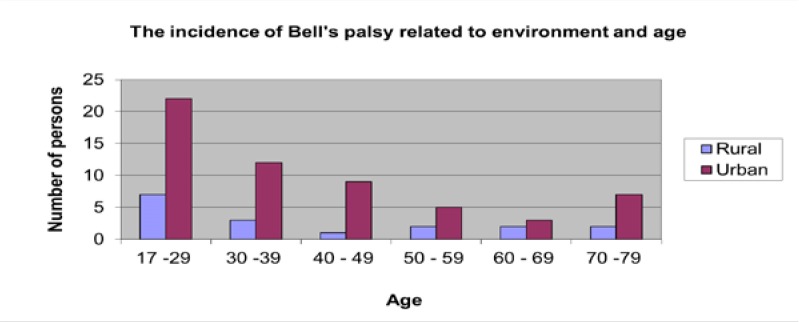
Incidence related to environment and age

**Fig. 5:**
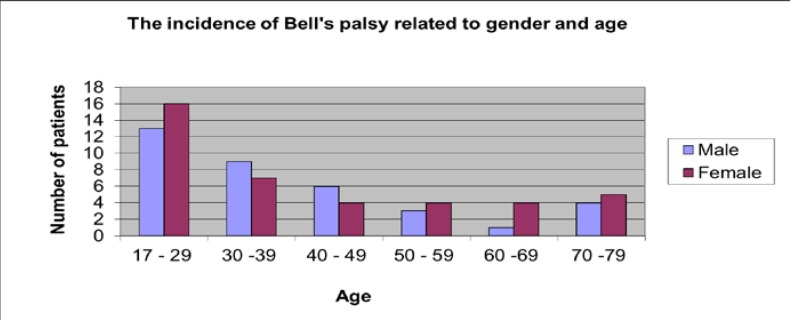
Incidence related to gender and age

Regarding the age distribution, the highest incidence was identified between 17 an 29 years old and the lowest incidence between the age of 60 to 69 (**Fig. 5**).

More patients from the urban area have been admitted (**Fig. 4**).

**Fig. 6:**
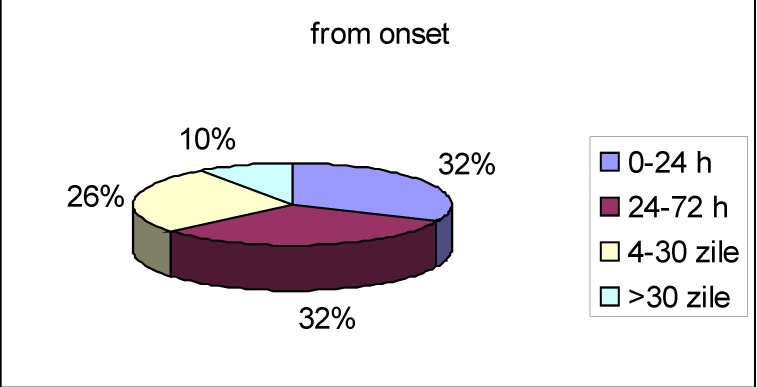
Time of onset

32 percent of the patients were admitted within 24 hours from the onset of the symptoms, another 32 percent between the first and the third day, and 26 percent came to the hospital within the first month (**Fig. 6**).

**Fig. 7:**
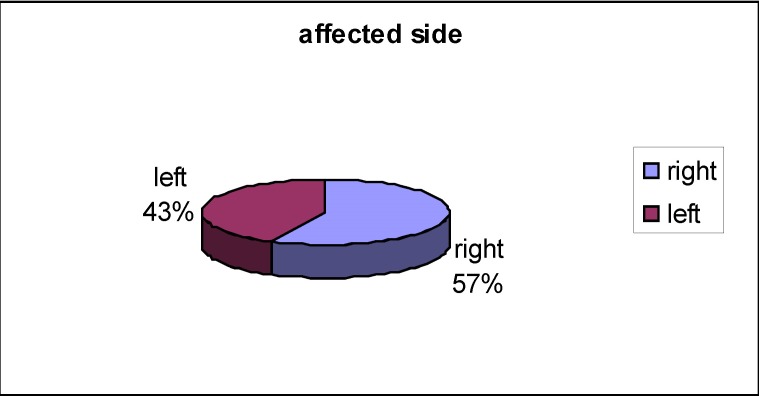
Affected face side

There was a predominance of females and the right side was more frequently affected in the studied population (**Fig. 7**).

Regarding the associated complains: 5 patients accused ipsilateral ear pain, one presented neurosensorial hearing loss to the ipsilateral ear, one had vertigo. Diabetes mellitus and high blood pressure were also found.

**Fig. 8:**
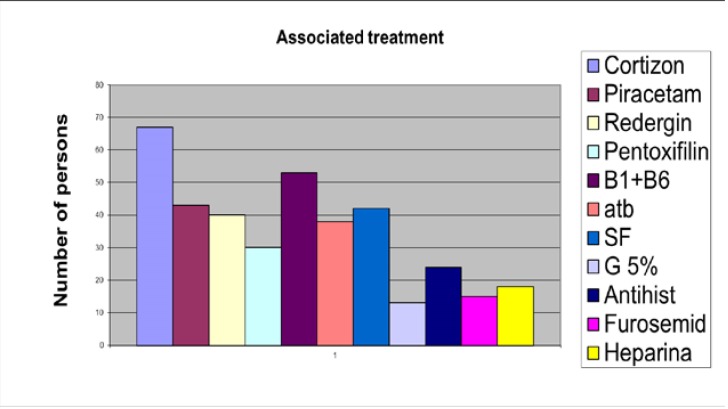
Drugs received

**Fig. 9:**
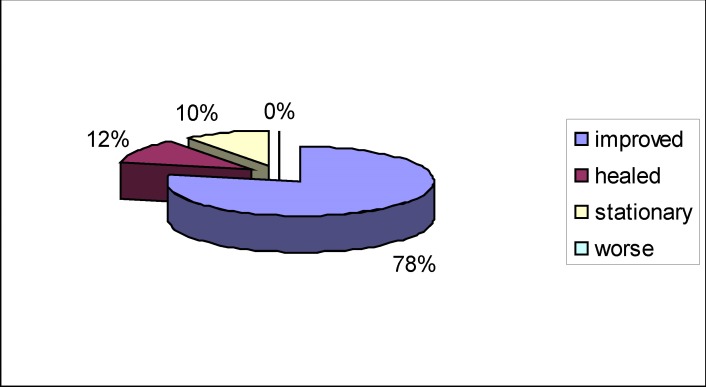
Results at discharge

The patients received treatment mainly with steroids, neurotrophic agents, vasodilatation agents, vitamins B (**Fig. 8**), with the results shown below, recorded when leaving the hospital (**Fig. 9**). The best results have been registered when therapy was initiated within three days from the onset, at partial paralysis.

## Discussions

Bell's palsy is a common but still controversial disease, with unknown etiology until now. Modern literature showed that up to 80 percent of the patients will recover without treatment [**2**], however, favorable outcome was recorded by using vasodilators, neurotrophic and corticosteroid therapy and the initiation of steroid treatment within three days from the onset of symptoms increased the chance of complete recovery. Our hope is that this study will help clinicians manage Bell’s palsy and prevent recurrences.

## Conclusions

Bell's palsy remains the most common cause of facial nerve paralysis.

Our study found a significant incidence of recurrent facial palsy and, in some cases, an association with ipsilateral otic complaints, facial pain or paraesthesia.

It appears more often in females aged 17 to 30 years old and the right side seems to be affected more often.
